# Optimization of HLA-B*27 ALLELE Genotyping by PCR-SSP

**DOI:** 10.6061/clinics/2020/e1840

**Published:** 2020-10-21

**Authors:** Fernanda Formaggi Lara-Armi, Jeane Eliete Laguila Visentainer, Hugo Vicentin Alves, Marco Antônio Rocha-Loures, Janisleya Silva Ferreira Neves, Cristiane Maria Colli, Quirino Alves de Lima, Ricardo Alberto Moliterno, Ana Maria Sell

**Affiliations:** I Programa de Pos-Graduacao em Biociencias e Fisiopatologia, Departamento de Analises Clinicas e Biomedicina, Universidade Estadual de Maringa, Maringa, Parana, BR; II Programa de Pos-Graduacao em Biociencias e Fisiopatologia, Departamento de Analises Clinicas e Biomedicina e Laboratorio de Imunogenetica, Departamento de Ciencias Basicas da Saude, Universidade Estadual de Maringa, Maringa, Parana, BR

**Keywords:** Spondyloarthropathies, Ankylosing Spondylitis, HLA-B27 Antigen, Polymerase Chain Reaction, Genotyping Techniques

## Abstract

**OBJECTIVES:**

HLA-B27 is strongly associated with ankylosing spondylitis (AS) and its presence helps to confirm AS diagnosis. Due to the high HLA polymorphism and the differentiated contribution of alleles and molecules encoded by them, HLA-B*27 allele identification is relevant in the clinical follow-up, diagnosis, and treatment of this spondyloarthropathy. Inexpensive genotyping techniques with high specificity and sensitivity are of great interest in histocompatibility laboratories. This work aimed to optimize HLA-B*27 genotyping by Polymerase Chain Reaction Sequence-specific Primer (PCR-SSP), which is an accessible and inexpensive technique.

**METHODS:**

The PCR-SSP was standardized using 26 HLA-B*27 positive and 3 HLA-B*27 negative samples previously defined by Polymerase Chain Reaction Sequence-specific Oligonucleotide Probes (PCR-SSOP) (medium resolution, One Lambda^®^) and primers described by Duangchanchot et al. (2009). For validating the technique, 397 samples were genotyped using PCR-SSP as well as PCR-SSOP.

**RESULTS:**

The PCR-SSP technique was standardized for identifying the alleles HLA-B*27:02, HLA-B*27:CAFRW (05/13/16/17/28/37/38/39/42), HLA-B*27:CAFRZ (08/26/40), HLA-B*27:09 and HLA-B*27:12, which were found in 90 positive samples (22.67%). There was 100% agreement between the two techniques for heterozygous samples; however, two homozygous samples could not be detected by PCR-SSP.

**CONCLUSION:**

The HLA-B*27 genotyping using PCR-SSP, an easy-to-use, specific, and affordable technique, was optimized for heterozygous samples. This technique may contribute to AS diagnosis.

## INTRODUCTION

Leukocyte antigen system (HLA), a major histocompatibility complex (MHC), is located on the short arm of chromosome 6 in humans (1). This system has an extensive polymorphism for class I and II genes, characterized by a high number of alleles. As of October 2019, 25,756 HLA alleles have been described, of which 18,691 are class I alleles and 7,065 are class II alleles. *HLA-B* is the most polymorphic locus of the HLA system, with 7,053 alleles identified (2). Class I human leukocyte antigens are expressed on most nucleated cell surfaces. They carry endogenous peptides to the cell surface for recognition by T-cell receptors and their functions are involved in immune responses. Thus, many diseases are associated with HLA (3).

Ankylosing spondylitis (AS), a chronic inflammatory disease of the spondyloarthropathy group, is strongly associated with the HLA-B27 antigen. This association was first described in 1973 and is the largest genetic factor contributing to the disease (4). Early genetic association studies in Caucasians showed that HLA-B*27 was present in approximately 90% of the individuals with AS. Further studies confirmed this association in other population, but with different strengths of association; thus, 50-90% of the individuals with this disease have the HLA-B*27 gene (5-7). The HLA-B*27 allelic group has more than 290 known alleles (8), although most alleles are not associated with AS. Studies have shown that HLA-B*27:05, HLA-B*27:04, and HLA-B*27:02 are associated with AS in the Caucasian, Chinese, and Mediterranean populations, respectively (4). The HLA-B*27:06 and HLA-B*27:09 alleles were not found to be associated with AS (4, 9, 10).

Inexpensive genotyping techniques with high specificity and sensitivity are of great interest in histocompatibility-testing laboratories. Duangchanchot et al. (2009) described specific primers for genotyping 42 HLA-B*27 alleles (B*27:01-B*27:21 and B*27:23-B*27:43) using high-resolution polymerase chain reaction with sequence-specific primer (PCR-SSP) (11). PCR-SSP has been reported to be a simple, fast, inexpensive, specific, and highly sensitive method (12).

The identification of the HLA-B*27 alleles is relevant in the clinical follow-up, diagnosis, and treatment of AS (9). This work aimed to optimize HLA-B*27 genotyping using PCR-SSP, an easy-to-use and affordable technique.

## MATERIALS AND METHODS

### Sample selection

Twenty-six samples previously known as HLA-B*27 positive and three HLA-B*27 negative samples using PCR-SSOP^^®^^ (medium resolution; One Lambda; Canoga Park, CA, USA) were collected from the UEM Immunogenetic Laboratory database (https://www.onelambda.com/en/products-services/products/molecular-typing/labtype.html) and used to standardize the method. For validation, samples were collected from the individuals with AS (N=160) and psoriatic arthritis (PsA; N=57) due to the high frequency of HLA-B*27 in this population; samples from individuals without the disease (N=180) were added to increase the randomness of the process. The patients with AS and PsA were classified through clinical, laboratory and radiological criteria according to the ASAS 2009/2011 criteria (13,14) and CASPAR (15), respectively, by rheumatologists from the Regional Maringá University Hospital. All participants were from the northwestern region of Paraná, southern Brazil (22°29′30--26°42′59- S and 48°2′24--54°37′38- W) and classified as ‘mixed ethnicity with predominantly European origin,’ based on the ethnic constitution of Paraná, as previously described (16) and confirmed for this region (17). The samples were collected sequentially from May 2014 to December 2016. This study was approved by the Research Ethics Committee of the State University of Maringá (UEM), number CAAE 27723114 and all participants signed the consent form.

### DNA extraction

DNA was extracted from whole blood or buffy coat collected in 5-mL tubes containing EDTA using the salting out method and/or DNA extraction kit BIOPUR^®^ (Mobius; Curitiba, Paraná, Brazil). DNA purity and concentrations were determined by NanoDrop^®^ 2000 UV-Vis spectrophotometer (Thermo Fisher; Wilmington, DE, USA). The concentration was adjusted to 50-100 ng/μL.

### Standardization of HLA-B*27 genotyping

The primers used for standardizing the PCR-SSP reaction were constructed according to the sequences described by Duangchanchot et al. (2009) and are shown in Table 1.

Two primer mixes (SC1 and SC2) were used to assess the presence of HLA-B*27 allelic group and nine mixes were used to identify the alleles. Primers amplifying a 782 bp fragment in the third intron of *HLA-DRB1* (C5: 5′-TGCCAAGTGGAGCACCCA-3′; C3: 5′- GCATCTTGCTCTGTGCAGAT-3′) were used as the internal control (18).

Same PCR conditions were used for all mixes (Table 2). The PCR mixture contained 1× standard *Taq* reaction buffer, 200 µM deoxyribonucleotide phosphates (dNTP), 2 ng/μL each specific primer, 1 ng/μL each internal control primer, and 40-200 ng template DNA in a 10-μL volume. The final concentrations of MgCl_2_ and *Taq* DNA polymerase were different in the mixes and are described in Table 3. PCRs were performed with a final DNA concentration of 40-200 ng, and the sensitivity for this DNA concentration range was same.

The PCR products were analyzed using 2% agarose gel electrophoresis stained with SYBR™ Safe DNA Gel Stain dye (Invitrogen; Carlsbad, CA, USA), after running at 100 V, 300 mA, 150 W for 20 minutes. Visualization and photo documentation were performed on the Quantum ST4 transilluminator (Vilber Lourmat; Collegien, France). Molecular weight markers with 100 base pairs (bp) (DNA Ladder, Thermo Fisher; Vilnius, Lithuania) was included to ensure the band sizes.

### Validation

For validating the technique, 397 samples were genotyped using the standardized PCR-SSP technique and confirmed using PCR-SSOP^^®^^, which is a routine method used in our laboratory (LIG-UEM). To avoid biased results, PCR-SSOP^^®^^ was performed after genotyping all samples using standardized PCR-SSP.

## RESULTS

The standardized PCR-SSP was performed using the same thermocycling conditions for all primers and a low final volume of reagents. Only the final concentrations of MgCl_2_ and *Taq* DNA polymerase were different for each primer mix used.

To validate the technique, 397 samples were genotyped using PCR-SSP and PCR-SSOP^^®^^. Using PCR-SSP, 90 samples (22.7%) were positive for mix SC1 and/or SC2, therefore being positive for HLA-B*27. The frequency of HLA-B*27 was 44.4%, 15.8%, and 5.5% in individuals with AS, PsA, and no spondyloarthropathy, respectively; the distribution of frequency was as expected for that observed in Brazilians (19-21) allowing to validate the technique. To define the HLA-B*27 alleles, these samples were genotyped with primer mixes 2, 3, 4, 5, 7, 8, 9, and 10. Mix 12 was used only when samples were positive after genotyping with mix 8. The genotyping results are shown in Table 4. HLA-B*27:02, HLA-B*27:02/HLA-B*27:CAFRW, HLA-B*27:CAFRW, HLA-B*27:CAFRZ, HLA-B*27:09, and HLA-B*27:12 alleles were identified in 2, 1, 84, 1, 1, and 1 individuals, respectively, using PCR-SSP. HLA-B*27:CAFRW allele frequency was significantly different between the patients with AS and PsA (21.6% and 7.0%, respectively) and individuals without spondyloarthropathies (2.8%).

After performing PCR-SSP, these same samples were genotyped using PCR-SSOP^^®^^. There was 100% agreement between the two techniques for the heterozygous samples. However, two homozygous HLA-B*27:CAFRW samples were not identified by standardized PCR-SSP (Table 4).

**Figure 1:**
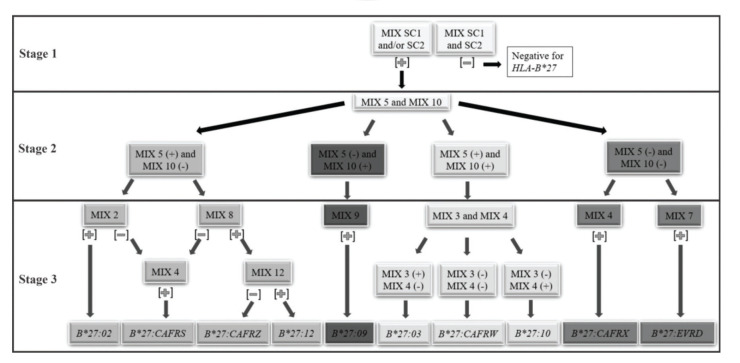
The reaction sequence used for genotyping the HLA-B*27 allelic variants. CAFRS: 04/15/25. CAFRZ: 08/26/40. CAFRW: 05/13/16/17/28/37/38/39/42. CAFRX: 06/18/20. EVRD: 07/34/43. (−) no band amplification or negative results. (+): band amplification or positive results.

A facilitator was proposed to define the reaction sequence for HLA-B*27 genotyping using the in-house PCR-SSP (Figure 1).

Thus, we proposed a short path for the definition of HLA-B*27 alleles and allelic variants. The reaction can be performed in three stages. **1st stage:** PCR-SSP with primer mixes SC1 and SC2. Positive samples for at least one of them define the positive HLA-B*27 genotype, which will be used for the next stage reactions. **2nd stage:** PCR-SSP with primer mixes 5 and 10. According to the results obtained, the different paths described in the next stage shall be used. **3rd stage:** *i.* mix 5 positive and mix 10 negative: PCR-SSP with primer mixes 2, 4, 8, and 12; *ii.* mix 5 negative and mix 10 positive: PCR-SSP with primer mixes 9; *iii.* both mix 5 and 10 positive: PCR-SSP with primer mixes 3 and 4; *iv.* both mix 5 and 10 negative: PCR-SSP with primer mixes 4 and 7. The third-stage reactions can be performed concurrently to facilitate the process. The results must be interpreted according to step 3 of the flowchart (Figure 1).

**Figure S1:**
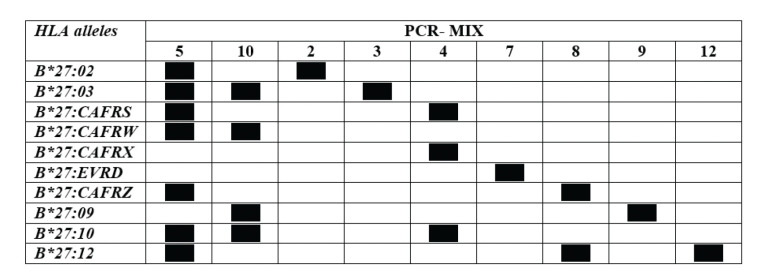
Worksheet for HLA-B*27 PCR-SSP reactions. *CAFRS: 04/15/25. CAFRW: 05/13/16/17/28/37/38/39/42. CAFRX: 06/18/20. EVRD: 07/34/43. CAFRZ: 08/26/40*. The positive reactions are marked with a black boxes.

**Figure S2:**
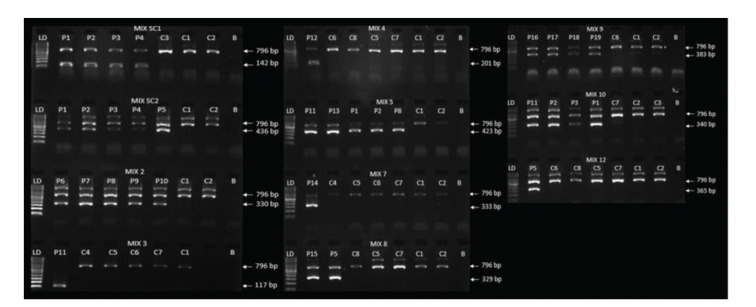
Band pattern of positive and negative samples for HLA-B*27 allelic variants for all standardized PCR-SSP. P: HLA-B*27 positive sample; C: HLA-B*27 negative sample; B: blank. Step ladder (LD): 100 base pairs (bp). Internal control: 796 bp. Band size of each mix: mix SC1: 142 bp; mix SC2: 436 bp; mix 2: 330 bp; mix 3: 117 bp; mix 4: 201 bp; mix 5: 423 bp; mix 7: 333 bp; mix 8: 329 bp; mix 9: 383 bp; mix 10: 340 bp; mix 12: 365 bp.

The worksheet for HLA-B*27 PCR-SSP and band patterns of positive and negative samples using standardized PCR-SSP are shown in Appendix figures S1 and S2, respectively.

## DISCUSSION

HLA-B*27 allelic genotyping has become important in clinical practice for the treatment and management of spondyloarthropathies. In AS, HLA-B*27 is the most important genetic marker and some alleles such as HLA-B*27:05, B*27:02, B*27:04, and B*27:07, have been associated with the disease (4,9). Through the standardized PCR-SSP technique, it was possible to genotype the HLA-B*27 alleles frequent in the Brazilian population. This technique can be used as an auxiliary method for diagnostic purposes, as well as in genetic association studies to estimate the HLA-B*27 frequency in a specific population.

To define the HLA-B*27 allele with minimal PCRs, a flow chart has been proposed (Figure 1). The first stage defines the positivity for the HLA-B*27 allelic group and the specific allele is identified in samples heterozygous for the *HLA-B* locus in two further steps. We consider the methodology of high resolution, although some alleles, such as HLA-B*27:05 more frequent in our population, could not be identified without some alleles with low frequencies.

**Figure S3:**
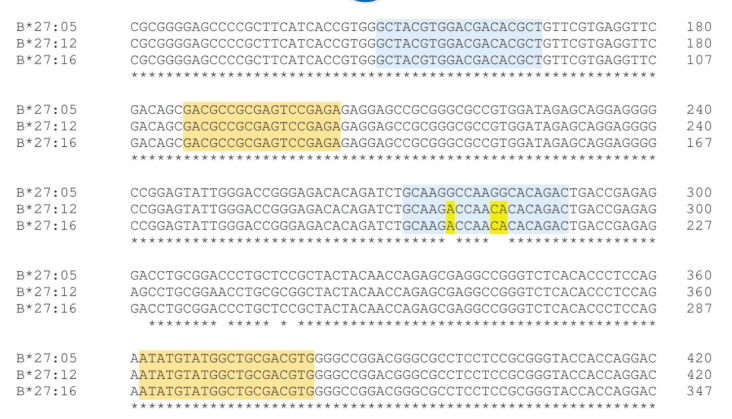
Clustal Omega multiple sequence alignment from the PCR-amplified regions of HLA-B*27:05, B*27:12, and B*27:16. The binding sites of SC1 and SC2 primer pairs are highlighted in light blue and orange, respectively. The yellow highlight indicates the divergent nucleotides.

**Figure S4:**
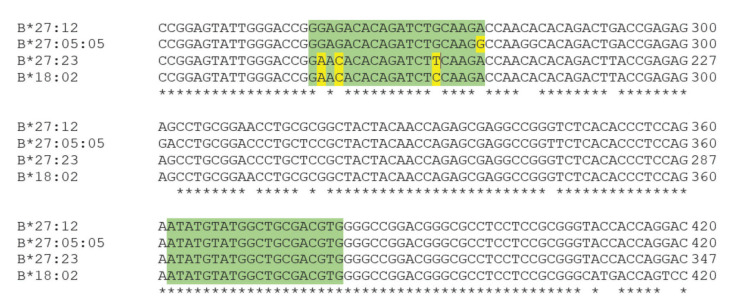
Clustal Omega multiple sequence alignment from the PCR-amplified regions of HLA-B*27:12, B*27:05:05, B*27:23, and B*18:02. The binding sites of mix 12 primer pairs are highlighted in green. The yellow highlights indicate the divergent nucleotides.

According to Duangchanchot et al. (2009), SC1 and SC2 mixes can be used to detect whether the samples are HLA-B*27-positive or -negative (11). Our results were in agreement with this, but there was a discordance for SC1 mix, which did not amplify HLA-B*27:12 and HLA-B*27:16 alleles. As shown in Appendix Figure S3, there are three nucleotides that diverge between the sequence of SC1 reverse primer and the sequence of HLA-B*27:12 and B*27:16 alleles. Since the PCR is performed at high annealing temperatures (68°C and 65°C) for almost the entire amplification process, the SC1 reverse primer is unable to bind to the template due to these mismatches, which can lead to inefficient or no amplification of those alleles. Appendix Figure S4 shows a similar situation for PCR-SSP with primer mix 12. In this situation, the alleles HLA-B*27:05:05, B*27:23, and B*18:02 have a low amplification efficiency and Duangchanchot et al. (2009) describes a possible amplification for these alleles.

PCR-SSP has been described as being more economical, relatively simple, fast, and highly sensitive and specific than other methods, (12,22). In our study, the standardized PCR-SSP technique showed 100% sensitivity and specificity for HLA-B heterozygous samples, as measured by the concordance of the PCR-SSP and PCR-SSOP results. The main advantage of this technique is that it can be performed in small laboratories with simple equipment, such as a thermal cycler, PCR workstation, and electrophoresis apparatus.

Rare alleles were not detected using the standardized PCR-SSP method and this limitation was due to the database used for standardizing the technique. Another limitation of the technique is that it cannot detect homozygous HLA-B*27 samples. The frequency of individuals homozygous for HLA-B*27 varies in different populations. Zou et al. (2015) identified one (0.4%) homozygous patient (HLA-B*27:05/27*05) among 247 Chinese patients with AS (23). Yi et al. (2013) studied 336 positive HLA-B*27 Korean patients with AS and identified 9 (2.67%) to be homozygous (24). In this study, we identified two (2.2%*)* homozygous individuals (HLA-B*27:CAFRW/*27:CAFRW) among 90 HLA-B*27 positive individuals. Studies have reported that individuals who were homozygous for HLA-B*27 are more susceptible to develop AS (5,25,26), but it was not linked to severe clinical manifestations of the disease (26-28). Other genotyping techniques, such as sequencing or high-resolution PCR-SSOP, should be performed to identify the homozygosis. In addition, to define heterozygosis in homozygous samples for the HLA-B*27 allelic variants, all PCR-SSPs (Figure 1) must be performed.

The standardized technique may be used as an auxiliary method in the diagnosis of AS and other diseases as well as in future genetic association studies.

## CONCLUSION

This study standardized and optimized a PCR-SSP method for HLA-B*27 genotyping in heterozygous individuals, which was considered of high definition, with good sensitivity and affordability.

## APPENDIX

### Supplementary Material

## AUTHOR CONTRIBUTIONS

Lara-Armi FF carried out the writing of the manuscript and performed the standardization and the validation of the PCR-SSP; Visentainer JE, Colli CM and Moliterno RA were responsible for the study design; Alves HV carried out the PCR-SSOP for validation; Rocha-Loures MA and Neves JS were responsible for sample collection; Lima Neto QA was responsible for in silico analyses. Sell AM was responsible for the study design, writing and revision of the manuscript.

## Figures and Tables

**Table 1:** Primers for genotyping HLA-B*27 (Mix SC1 and SC2) and specific alleles (Mix 2, 3, 4, 5, 7, 8, 9, 10, 12).

Mix	Name	P	Sequence (5′ - 3′)	Location	Position	HLA-B*27 alleles amplified
SC1	F167T	1	GCT ACG TGG ACG ACA CGC T	Exon 2	149-167	01-11, 13-15, 17, 19-21,
	R272G	2	GTC TGT GCC TTG GCC TTG C	Exon 2	272-290	24-28, 30-43
SC2	F204A	3	GAC GCC GCG AGT CCG AGA	Exon 2	187-204	01-06, 08-10, 12-13, 15-18,
	R362A	4	CAC GTC GCA GCC ATA CAT AT	Exon 3	362-381	20, 23, 25-29, 31, 35-42
2	F311T	6	ACC GAG AGA ACC TGC GGA T	Exon 2	293-311	02
	R362A	4	CAC GTC GCA GCC ATA CAT AT	Exon 3	362-381	
3	F167T	7	GCT ACG TGG ACG ACA CGC T	Exon 2	149-167	03
	R247C	8	GTG TCT CCC GGT CCC AAT G	Exon 2	247-265	
4	F362A	9	GGT CTC ACA CCC TCC AGA A	Exon 3	344-362	04, 06, 10, 15, 18, 20, 25
	R527A	10	CTC TCA GCT GCT CCG CCT	Exon 3	527-544	
5	F272G	11	ACC GGG AGA CAC AGA TCT G	Exon 2	254-272	01-05, 08, 10, 12-17, 19, 25-26
	R418G	12	CTT GCC GTC GTA GGC GTC	Exon 3	418-434	28, 30-32, 36-40, 42
7	F301G	14	GCA CAG ACT GAC CGA GAG G	Exon 2	283-301	07, 32, 34, 43, B*0727,
	R363C	15	CAC GTC GCA GCC GTA CAT G	Exon 3	363-381	B*3707, B*3709
8	F311A	16	CCG AGA GAG CCT GCG GAA	Exon 2	294-311	08, 12, 18, 26, 40, 42, B*1802
	R362A	4	CAC GTC GCA GCC ATA CAT AT	Exon 3	362-381	
9	F272G	11	ACC GGG AGA CAC AGA TCT G	Exon 2	254-272	09
	R418C	17	CTT GCC GTC GTA GGC GTG	Exon 3	418-434	
10	F301G	14	GCA CAG ACT GAC CGA GAG G	Exon 2	283-301	03, 05, 09-10, 13, 16-17, 27-29
	R362A	4	CAC GTC GCA GCC ATA CAT AT	Exon 3	362-381	35, 37-39, 41-42,
						B*3702, B*4701, B*4705
12	F277A	19	GGA GAC ACA GAT CTG CAA GA	Exon 2	258-277	12, 16, 18, 29,
	R362A	4	CAC GTC GCA GCC ATA CAT AT	Exon 3	362-381	B*3702, B*4704-05

Source: Adapted from Duangchanchot et al. (2009). P: primer identification.

**Table 2:** Thermocycling conditions for the HLA-B*27 genotyping using polymerase chain reaction with sequence-specific primer (PCR-SSP).

Cycle	Denaturation	Annealing	Extention
1 Cycle	96°C; 2 min	—	—
5 Cycles	96°C-30 s	68°C-60 s	72°C-40 s
21 Cycles	96°C-30 s	65°C-60 s	72°C-40 s
4 Cycles	96°C-30 s	55°C-75 s	72°C-120 s
1 Cycle	—	—	72°C; 10 min

Min: minutes. s: seconds.

**Table 3:** Concentration of reagents for different primer mixes used to define the HLA-B*27 allelic variants and the expected amplified fragment size.

MIX	Primer Identification	MgCl_2_ (mM)	*Taq* DNA Polymerase (U)	Band size
SC1	1 and 2	1.5	0.5	142
SC2	3 and 4	2.0	0.5	436
2	6 and 4	3.0	1.0	330
3	7 and 8	1.5	0.5	117
4	9 and 10	1.5	0.8	201
5	11 and 12	1.5	0.8	423
7	14 and 15	1.5	0.8	333
8	16 and 4	1.5	0.8	329
9	11 and 17	2.0	0.5	383
10	14 and 4	1.5	0.8	340
12	19 and 4	2.0	0.8	365

Primer identifications are described in Table 1.

**Table 4:** Number and definition of HLA-B*27 alleles identified using PCR-SSP and PCR-SSOP^^®^^.

	PCR-SSP N=397	PCR-SSOP^^®^^ N=397
HLA-B*27 negative	307	307
HLA-B*27 positive	90	90
HLA-B*27:02	2	2
HLA-B*27:CAFRW	84	82
HLA-B*27:CAFRZ	1	1
HLA-B*27:09	1	1
HLA-B*27:12	1	1
HLA-B*27:02/B*27:CAFRW	1	1
HLA-B*27:CAFRWB*27:CAFRW	0	2

N: number of individuals. CAFRW: 05/13/16/17/28/37/38/39/42. CAFRZ: 08/26/40.

## APPENDIX

Supplementary Material
